# The Value of SIRT1/FOXO1 Signaling Pathway in Early Detection of Cardiovascular Risk in Children with β-Thalassemia Major

**DOI:** 10.3390/biomedicines10102601

**Published:** 2022-10-17

**Authors:** Hoda A. Ibrahim, Soha S. Zakaria, Manal M. El-Batch, Mohamed R. El-Shanshory, Zahrah R. Alrayes, Ahmed M. Kabel, Samia A. Eldardiry

**Affiliations:** 1 Medical Biochemistry Department, Faculty of Medicine, Tanta University, Tanta 31527, Egypt; 2 Medical Biochemistry Department, Imam Muhammad Ibn Saud Islamic University, Riyadh 11564, Saudi Arabia; 3 Pediatric Department, Faculty of Medicine, Tanta University, Tanta 31527, Egypt; 4 Department of Biology, College of Science, Jouf University, Sakaka 2014, Saudi Arabia; 5 Department of Pharmacology, Faculty of Medicine, Tanta University, Tanta 31527, Egypt

**Keywords:** β-thalassemia major, carotid artery intima media thickness, β-stiffness index, SIRT1, FOXO1

## Abstract

*Background:* Atherosclerosis represents one of the major causes of morbidity in children with β-thalassemia major (β-TM). *Aim:* This study was designed to investigate SIRT1-FOXO1 signaling in β-TM children and their role in early detection of premature atherosclerosis. *Methods:* We equally subdivided 100 Egyptian children aged 6–14 years with β-TM according to carotid intima media thickness (CIMT) into 50 with CIMT < 0.5 mm and 50 with CIMT ≥ 0.5 mm, and 50 healthy children of matched age were included. They were subjected to evaluation of SIRT1, heat shock protein 72 (HSP72), and hepcidin levels via ELISA and forkhead box protein 1 (FOXO1) mRNA expression using real-time PCR in PBMCs; meanwhile, malondialdehyde (MDA), superoxide dismutase (SOD), and catalase activities were evaluated spectrophotometrically. *Results:* Our results show significantly high values for CIMT, β-stiffness, atherogenic index of plasma (AIP), MDA, HSP72 and FOXO1, ferritin with significantly low hepcidin, SOD, catalase, and SIRT1 in β-TM as compared to controls with a more significant difference in β-TM with CIMT ≥ 0.5 mm than those with CIMT < 0.5 mm. A significant positive correlation between CIMT and MDA, HSP72, and FOXO1 gene expression was found, while a significant negative correlation with hepcidin, SOD, catalase, and SIRT1 was found. FOXO1 gene expression and HSP72 levels were the strongest independent determinants of CIMT. *Conclusion:* In β-TM, FOXO1 signaling is activated with low levels of SIRT1, and this is attributed to accelerated atherosclerosis in β-TM, which would be crucial in prediction of atherosclerosis.

## 1. Introduction

Beta-thalassemia major (β-TM) is an autosomal recessive genetic anemia caused by a reduction in the globin chain formation leading to an imbalance between β-globin and non-globin chains, causing defects in the production of hemoglobin, which affects several organs and is associated with significant morbidity and mortality [1]. In erythroid cells, the β-globin tetramers precipitate and aggregate, creating inclusion bodies attached to the cell membrane. These inclusion bodies cause oxidative membrane damage and premature apoptotic death of erythroid precursors in the bone marrow, resulting in inefficient erythropoiesis [2]. In patients with β-TM, there is systemic iron overload. Hepcidin, the major iron-regulating hormone, is downregulated by anemia and hypoxia, causing increased duodenal iron absorption and resulting in systemic iron overload. Additionally, the disintegration of red blood cells causes iron overload in regularly transfused patients [3]. Iron excess causes changes in the arterial anatomy and carotid artery thickness, which acquire clinical indications of iron overload, including heart malfunction and left ventricular failure. Therefore, patients on a frequent transfusion regimen should be sufficiently chelated [4]. Children suffering from β-TM are considered as potentially suitable candidates for the ongoing study to detect early vascular changes, as atherosclerosis and coronary heart disease have emerged as important cardiovascular complications among β-TM patients [5,6,7]. β-TM is associated with an increased risk of atherosclerosis and related cardiovascular disease [8]. The mechanisms underlying accelerated atherosclerosis in β-TM were not clear up till now because the traditional risk factors fail to account fully for the excess of cardiovascular events in β-TM patients [9]. Therefore, it has been suggested that patients with β-TM possess additional risks in addition to the traditional risk factors for the development of accelerated atherosclerosis [10]. Patients with β-TM exhibit a higher increased carotid intima medium thickness (CIMT), which is a hallmark of early carotid atherosclerosis. CIMT acts as a structural indicator for early atherosclerosis in β-TM children and is evaluated via Doppler ultrasonography as a diagnostic tool [5]. There is a strong correlation between coronary atherosclerosis and the carotid arteries. Carotid ultrasound has been used for cardiovascular risk stratification in the general population. Intimal-medial thickness (IMT) is a non-invasive predictor of early arterial wall alteration, which identifies and quantifies early structural vascular abnormalities and is currently considered a marker of premature atherosclerosis [11]. 

Forehead box protein 1 (FOXO1) is the fundamental transcription factor that governs the expression of many cell cycle regulators; it is implicated in redox signaling as well as multiple atherogenic pathways in endothelial cells and is highly expressed in atherosclerotic plaques [12]. FOXO1 activity is regulated by Akt acetylation at several lysine sites and deacetylation by several deacetylases, notably the NAD + -dependent deacetylase SIRT1 in response to oxidative stress and hyperglycemia [13]. During oxidative stress, the cells may express a class of molecular chaperones called heat shock proteins to protect against proteotoxicity. FOXO appears to work in tandem with the heat shock proteins to induce transcription of both small and big heat shock protein genes, according to several studies [14,15]. This gene is thought to protect cells against protein misfolding caused by oxidative stress [14]. Furthermore, heat shock factor 1 binds to the promoters of heat shock protein (HSP) genes via SIRT1-mediated deacetylation, resulting in enhanced expression of the molecular chaperones HSP70 and HSP72 [16].

SIRT1 (sirtuin1) is abundantly expressed in vascular endothelial cells, particularly microvascular endothelial cells; it has been demonstrated to influence endothelial activities and vascular tone by deacetylating and activating endothelial nitric oxide synthase [17]. In addition, it has anti-inflammatory functions in endothelial cells and macrophages by suppressing the expression of endothelial tissue factors and downregulating the expression of various pro-inflammatory cytokines, and so it appears to have a pivotal role in cardioprotection [18].Therefore, the present study was designed to innovatively evaluate sub-clinical atherosclerosis in β-TM children via the incorporation of CIMT and the β stiffness index as well as SIRT1, HSP72, MDA, SOD, and catalase, along with FOXO1 gene expression, to gain biological insight on the molecular mechanisms of premature atherosclerosis, which may be beneficial in the early prediction and treatment of cardiovascular complications.

## 2. Materials and Methods

### 2.1. Inclusion and Exclusion Criteria

This study was carried out on 100 β-thalassemia major children aged 6–14 years presented to the Outpatient Clinic of the hematology unit of the Pediatric Department, Tanta University Hospital, Tanta, Egypt. as Additionally, 50 healthy children of matched age and sex represented the control group (Group I). β-thalassemia major was divided according to carotid intima-media thickness (CIMT) into Group II with CIMT ≤ 0.5 (*n* = 50) and Group III with CIMT > 0.5 (*n* = 50). All eligible children (patients and controls) were subjected to: history taking with special emphasis on demographic characteristics, disease duration, frequency of transfusions, and iron chelation regimen including type, dose, duration, and compliance. Thorough clinical examination including anthropometric measures and all system evaluations were performed. Routine laboratory investigations for follow-up thalassemia patients that included complete blood count, liver function tests, renal function tests, plasma ferritin, C-reactive proteins, CPK-MB, ANP, LDH, and hepatitis markers were taken.

Exclusion criteria include those with familial hypercholesterolemia (confirmed by history), chronic systemic illness, renal failure, hepatitis C virus infection, liver failure, diabetes mellitus, chronic hepatitis, and HIV infection. Informed written consent was obtained from the parents of all children enrolled in the study. 

### 2.2. Echocardiography

This was carried out via Duplex Ultrasound B-mode and color-coded duplex sonography. All studies were performed using Tissue Doppler using a G.E. Vivid 7 echocardiogram. All ultrasound examinations were performed by a pediatric cardiologist who was unaware of the clinical and laboratory details of the examined children.

#### 2.2.1. Carotid Intima-Media Thickness (CIMT)

The common carotid artery in the lower neck in the transverse plane was evaluated for the presence of subintimal lucency and atherosclerotic plaques that bulge into the lumen, followed by measuring the intimal plus medial thickness (IMT) [19]. For each subject, three measurements on both sides were obtained on the anterior, lateral, and posterior projection of the far wall. Values for the different projections and the right and left arteries were then averaged. The mean CIMT value for each subject was calculated using the formula [(left IMT+ right IMT)/2] and this value was taken as the measure of current common carotid artery wall thickness [20].

#### 2.2.2. Arterial Carotid Beta Stiffness (β Index)

To determine the stiffness of the vessel wall independent of changes in carotid artery pressure, the β-stiffness index was calculated [5,21]. Arterial stiffness was measured and calculated by selecting and averaging five consecutive waves and calculated using the following established formula:

β = 1n(SBP/DBP)/[(Ds-Dd)/Dd]

where SBP is (the systolic blood pressure), DBP is (the diastolic blood pressure), and Ds and Dd are the (maximum and minimum CCA diameters) respectively. The blood pressure was measured using a conventional digital blood pressure monitor with an upper arm cuff method. Additionally, the effect of breathing was taken into consideration [5,21].

### 2.3. Blood Sample Collection 

Venous blood samples were obtained from all subjects after overnight fasting. Of the samples, 2 mL of blood was placed in a heparin vacutainer tube for genetic study and the rest of the samples were collected in a disposable plastic tube containing EDTA, centrifuged for plasma separation, and immediately frozen at −80 °C for future analysis.

### 2.4. Biochemical Assessment

#### 2.4.1. Assessment of SIRT1, FOXO1, Hepcidin, and HSP72

Quantitative determination of SIRT1 (CAT#ab171573), FOXO1(CAT# ab215087), (supplied by Abcam, USA), hepcidin (CAT#A3770) and HSP72 (CAT# A76715) supplied by antibodies.com, accessed on 17 September 2022, UK) levels using an enzyme-linked immunosorbent assay (ELISA) was performed with commercially available kits according to the manufacturer’s instructions. Concentrations were calculated using a standard curve generated with specific standards provided by the manufacturer. 

#### 2.4.2. Assessment of Redox Status Parameters

Spectrophotometric assay of catalase, superoxide dismutase (SOD) (CAT# SD 25 21), and malondialdehyde (MDA) (CAT# MD 25 29) levels using commercial kits (Bio-diagnostic, Giza, Egypt).

#### 2.4.3. Determination of FOXO1 Gene Expression

Expression of FOXO1 gene in peripheral blood mononuclear cells (PBMCs) was detected by using real-time PCR (RT-PCR). PBMCs were prepared via density gradient centrifugation using Ficoll-Hypaque (Thermo Scientific, USA). Briefly, heparinized blood was carefully layered on Ficoll, and PBMC was harvested from the white interphase after centrifugation for 30 min at 400 g at room temperature and washed with phosphate-buffered saline (PBS) [22]. The PBMC samples were stored at −80 °C. The samples were further processed for RNA isolation according to the manufacturer’s protocol using the Gene JET RNA Purification Kit (Thermo Scientific, USA). Total RNA was reverse transcribed using Revert Aid H Minus Reverse Transcriptase (Thermo Scientific, USA) to produce cDNA to be used as a template. PCR reactions were performed using Power SYBR Green PCR Master Mix (Life Technologies, Carlsbad, CA, USA) following the manufacturer’s instructions. This cDNA was then amplified using the Step One instrument (Applied Biosystems, USA) as follows: Initial denaturation at 95 °C for 5 min was followed by 30 cycles with denaturation at 95 °C for 30 s, annealing at 60 °C for 30 s, and extension at 72 °C for 30 s. A control reaction without a DNA template was performed in parallel to detect genomic DNA contamination. FOXO1 mRNA transcripts were quantified relative to the housekeeping gene GAPDH, which was used as an internal control. Sequence-specific primers were designed as follows: FOXO1 forward 5′-AACTTTCGCTTAGTGGAACGT-3′, FOXO1 reverse: 5′-ACCCTCATACCTTTGGAACAG-3′. The housekeeping gene GAPDH (with primer sequences; forward: 5′ ATGACATCAAGAAGGTGGTG -3′ and reverse: 5′-CATACCAGGAAAATGAGCTTG-3′) [23]. The determination of the relative levels of gene expression was automatically calculated using the comparative threshold (∆∆ Ct) method and normalized to the reference gene, GAPDH, which was not altered by the experimental conditions [24]. 

### 2.5. Statistical Analysis 

Analysis of the results of this study was performed using the computer SPSS program used for all statistical calculations (Statistical Package for the Social Science; SPSS, Chicago, IL, USA) version 24 for Microsoft Windows, USA. Statistical analysis of the present study was conducted for quantitative variables, which were expressed as means ± SD. An analysis of variance (ANOVA) test followed by post hoc analysis was used for multiple comparisons between different groups. A Pearson correlation was used to detect the relationship between CIMT and different parameters. Partial correlation was used to determine the relation between CIMT and each biomarker independent to the effect of other studied parameters. Associations between CIMT and other risk factors were evaluated by multiple linear regressions. The receiver operating characteristic (ROC) curve was performed for the optimized cut-off points detection for SIRT1 and relative FOXO1 mRNA expression to reach the best compromise for pre-mature atherosclerosis in β-TM. The best cut-off values were based on the calculation of the Youden index, which is Maximum = Sensitivity + Specificity − 1. All statistical tests were two-tailed and only a *p* value ≤0.05 was considered statistically significant.

## 3. Results

Table 1 shows the clinical and laboratory characteristics of the studied groups. It shows a significant increase in values of CIMT, beta stiffness, AIP, MDA, ANP, LDH, HSP7, FOXO1, and FOXO1 relative gene expression in β-TM patients with CIMT more than 0.5 mm (group III) as compared to β-TM patients with CIMT less than 0.5 mm (group II) and the control group *p*-value < 0.05. Additionally, values of ferritin were higher in both thalassemic groups with a significant increase in β-TM patients with CIMT more than 0.5 mm, *p*-value < 0.05. However, there was a significant reduction of hepcidin, SOD, catalase and SIRT1 levels in both thalassemic groups as compared to controls with lower values in β-TM patients with CIMT of more than 0.5 mm, *p*-value < 0.05. 

Table 2 shows correlation analysis between mean CIMT and βstiffness and atherosclerotic risk factors in β-TM patients. It shows a significant positive correlation between CIMT with MDA, HSP72, ANP, LDH, FOXO1, and FOXO1 gene expression but a significant negative correlation with hepcidin, SOD, catalase, and SIRT1. Partial correlation shows a significant correlation between FOXO1 and HSP 72 with CIMT.

In Table 3’s multiple regression analysis, FOXO1 protein level and its gene expression as well as HSP72 levels were the strongest independent determinants of CIMT, *p*-value < 0.05.

**Figure 1:**
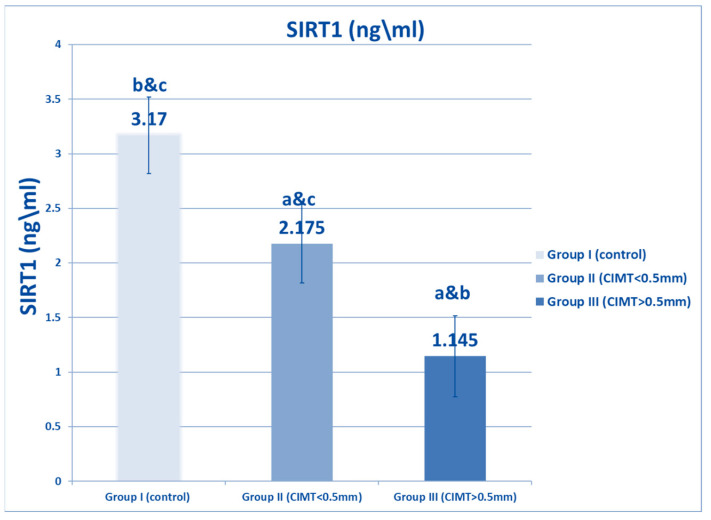
Comparison between studied groups with regard to SIRT1 level. Data are represented as mean ± SD (*n* = 50 in each group). Statistical analysis was carried out using one-way ANOVA with Tukey’s post hoc test, SPSS computer program. a–c: Significant difference between groups at *p* < 0.05. a: significance from Group I; b: significance from Group II; c: significance from Group III. SIRT1: sirtuin1.

**Figure 2:**
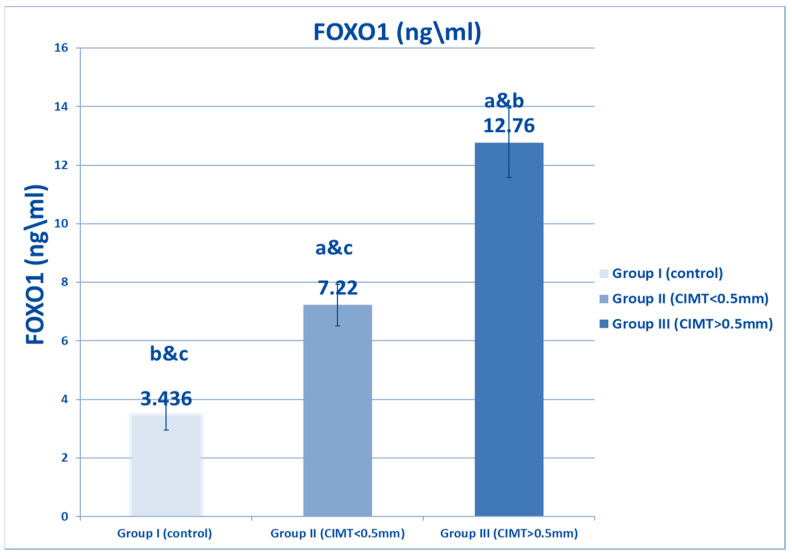
Comparison between studied groups with regard to FOXO1 level. Data are represented as mean ± SD (*n* = 50 in each group). Statistical analysis was carried out using one-way ANOVA with Tukey’s post hoc test, SPSS computer program. a–c: Significant difference between groups at *p* < 0.05. a: significance from Group I; b: significance from Group II; c: significance from Group III. FOXO1: forehead box protein 1.

**Figure 3:**
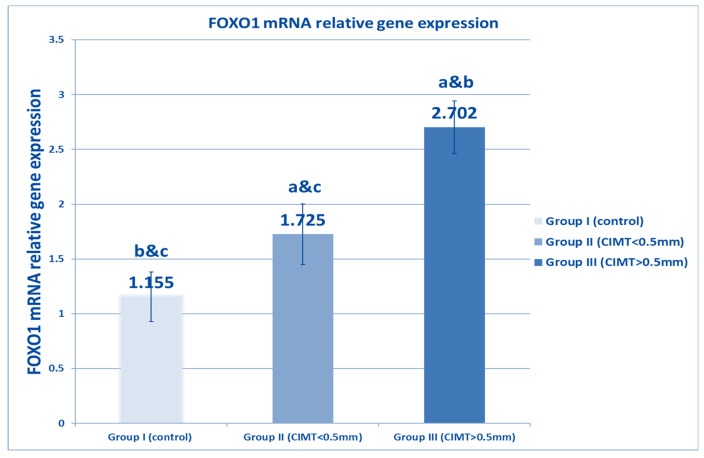
Comparison between studied groups with regard to FOXO1 mRNA relative to the gene expression level. Data are represented as mean ± SD (*n* = 50 in each group). Statistical analysis was carried out using one-way ANOVA with Tukey’s post hoc test, SPSS computer program. a–c: Significant difference between groups at *p* < 0.05. a: significance from Group I; b: significance from Group II; c: significance from Group III. FOXO1: forehead box protein 1.

Figure 1 shows that plasma SIRT1 levels were decreased in both β TM groups, and that there was a significant decrement in the β-TM group with CIMT of more than 0.5 mm (Group III) compared to the β TM group with CIMT of less than 0.5 (Group II), as well as to the healthy control (Group I). Figure 2 and Figure 3 show a significant increase in FOXO1 levels and FOXO1 relative gene expression in β TM with CIMT of more than 0.5 mm compared to other groups, *p* < 0.05.

**Figure 4:**
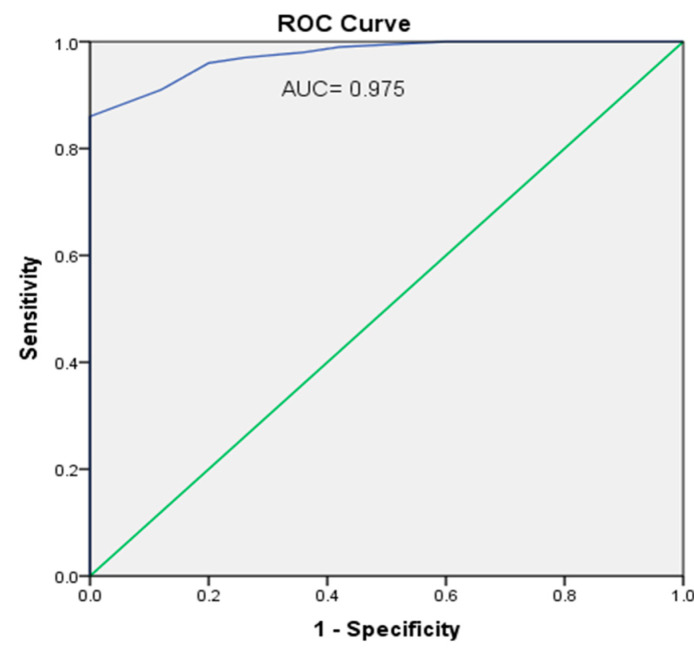
ROC curve shows area under the curve (AUC = 0.975). The optimal cut-off point for a relative SIRT1 level was 2.55 with a sensitivity of 91%, a specificity of 88% and Youden index = 178.

**Figure 5:**
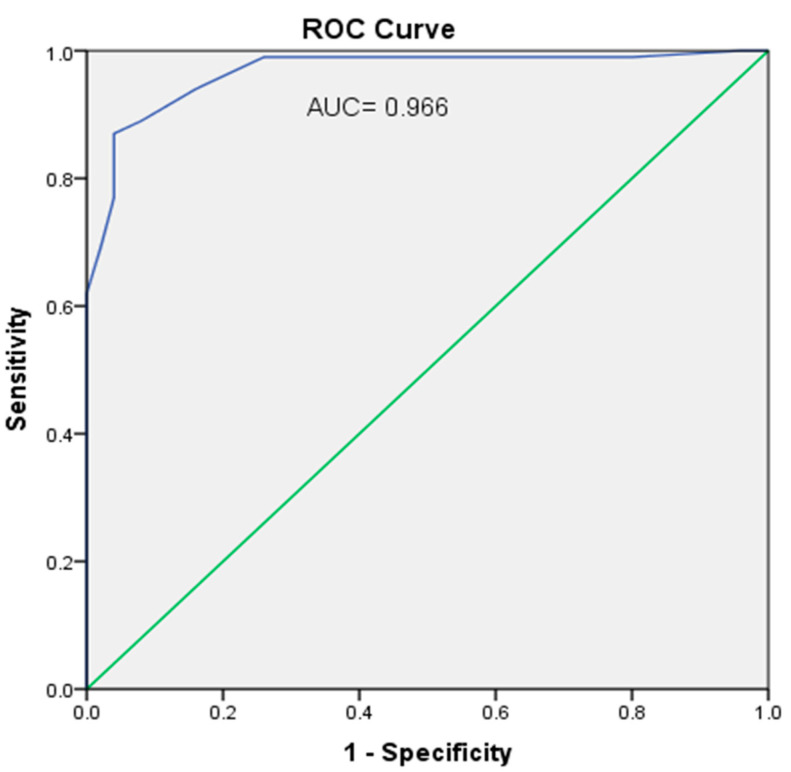
ROC curve shows area under the curve (AUC = 0.966). The optimal cut-off point for relative FOXO1 gene expression was 1.35 with a sensitivity of 94%, specificity of 84% and Youden index = 177.

Figure 4, the ROC curve for SIRT1, shows an optimal cut-off point, 2.55, with a sensitivity of 91%, specificity of 88% and Youden’s index = 178. Figure 5, the ROC curve for FOXO1 relative gene expression, shows the optimal cut-off point of 1.35 with a sensitivity of 94%, specificity of 84% and Youden’s index = 177.

**Figure 6:**
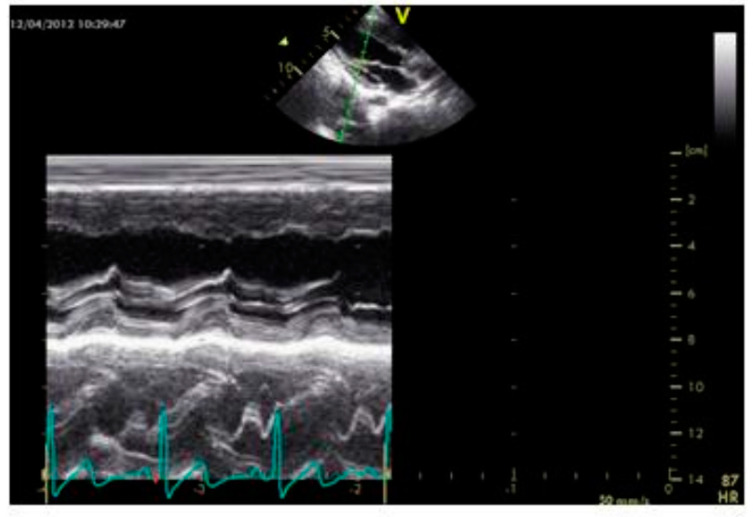
Showed M mode echocardiogram in β thalassemia major child.

**Figure 7:**
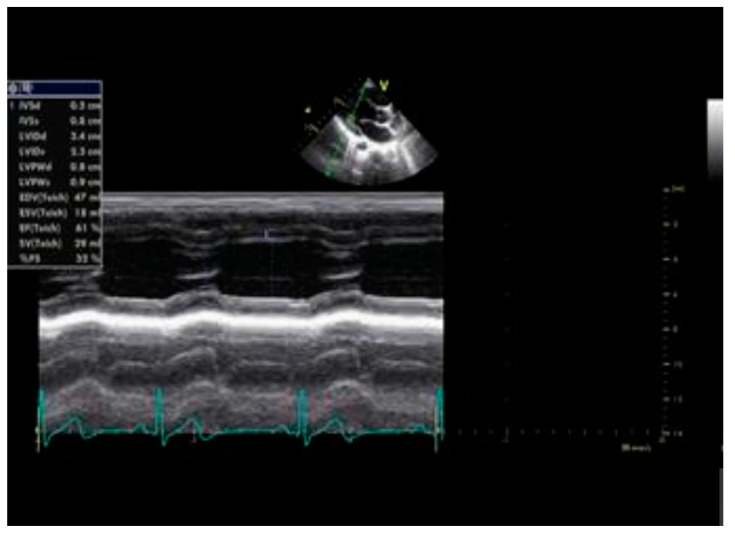
Shows M mode echocardiogram in a healthy child.

Figure 6 shows systolic and diastolic wall displacement of about four cycles on average in β thalassemia major children as compared to healthy children in Figure 7.

## 4. Discussion

In recent years, substantial scientific evidence has highlighted the detrimental impact of premature atherosclerosis, which is one of the major causes of morbidity and mortality in β-TM patients [6,25]. Our study revealed that in patients with β-TM, an increment in FOXO1 and HSP72 signaling with low levels of SIRT1 is linked to accelerated atherosclerosis, which is crucial as a novel biomarker for the prediction of atherosclerosis.

The complicated combination between conventional risk factors, iron overload, and its impact on oxidative stress may be the cause of the existence of accelerated atherosclerosis in β-TM [26]. Measurement of CIMT has been utilized in various research as a predictor of early atherosclerosis, as it is a noninvasive and early diagnostic method. CIMT has been revealed to predict both fatal and non-fatal cerebro- and cardio-vascular events and to significantly correlate with the existence of coronary artery disease (CAD) [27]. The current study demonstrates that CIMT is significantly higher among patients with β-TM than in control subjects, in alignment with previous reports [5,27,28]. Hemolytic anemia may be a risk factor for atherosclerosis through a variety of processes, while the actual cause of atherosclerosis is still unknown. First, during hemolysis, erythrocytes release the enzyme arginase alongside cell-free hemoglobin [29]. A low arginine–ornithine ratio contributes to dysregulated arginine metabolism by affecting the bioavailability of nitric oxide (NO), as these metabolic abnormalities reduce the amount of arginine that is available to nitric oxide synthase and cause vascular dysfunction [30]. Furthermore, since it has a deleterious impact on extra-hepatic lipolytic activity, anemia has been identified as a risk factor for hypertriglyceridemia [31]. Significantly high plasma ferritin was found among B-TM patients in our study; this conclusion was consistent with those of many earlier researchers [32,33]. Iron overload was affected by a combination of crucial and presumably correctable factors, including poor compliance with iron chelation. By generating several free radicals, a high iron burden may make the patient more vulnerable to atherosclerosis [34]. Moreover, a positive correlation between plasma ferritin and triglyceride level, “an important predictor of atherosclerosis”, was documented in previous work according to Ibrahim H A et al. [35]. Another study stated that non-transferrin-bound iron buildup at the cellular level and subsequent activation of macrophages may be the cause of atherosclerosis rather than elevated blood ferritin levels [36].

In agreement with Porter et al. [37], the current study shows a significant increase in plasma ferritin levels in both groups of β-TM as compared to controls. The excess iron determined in β-TM patients saturates the ability of the transferrin iron transport system, leading to non-transferrin-bound iron [37]. Iron overload promotes cellular malfunction, apoptosis, and necrosis by generating reactive oxygen species, which are produced by the metabolism of non-transferrin-bound iron [34]. Hepcidin is a hepatic peptide hormone that regulates iron homeostasis. Additionally, in accordance with previous reports, we discovered a significant reduction in hepcidin levels in both β-TM patients as compared to healthy children. Dietary iron absorption, plasma iron levels, and tissue iron distribution are all regulated by hepcidin. In iron-loading anemias such as β-thalassemia, hepcidin insufficiency is the primary or contributory factor of iron overload [38]. Hepcidin deficiency results from a strong suppressive effect of the high erythropoietic activity on hepcidin expression. Although in β-TM patients iron absorption contributes less to the total iron load than transfusions, in non-transfused thalassemia, low hepcidin and the consequent hyperabsorption of dietary iron is the major cause of systemic iron overload. Therefore, evaluating hepcidin concentrations alongside ferritin in those with iron-loading anemias may help identify those who are susceptible to experiencing iron toxicity since their hepcidin levels are so drastically low [38,39].

In the present study, not only was there a significant increase in CIMT and β arterial stiffness in Group III (CIMT more than 0.5 mm) as compared with Group II (CIMT less than 0.5 mm) and controls, but a significant correlation with body mass index and atherogenic index of plasma (AIP) was also detected, in agreement with Sherief et al. [7]. It thereby reflected our main concern to investigate the associated mechanisms identifying the relationship between AIP, plasma ferritin increments with CIMT, and β arterial stiffness in β-TM. Moreover, their co-link to assess changes in SIRT1, HSP 72, and FOXO1 and its relative gene expression in response to oxidative stress was investigated in relevance to changes in SOD and catalase levels in agreement with Kobayashi et al. [40].

In the current study, there was a significant increase in the AIP monitored in Group III (CIMT more than 0.5 mm) when compared to Group II (CIMT less than 0.5 mm) and the control group. The AIP is a marker of atherogenicity since it is related directly to atherosclerosis; it is calculated as log (TG/HDL-C). Hypertriglyceridemia will increase the activity of hepatic lipase, which results in HDL-C degradation with subsequent increased risk of coronary atherosclerosis [41]. In the present work, there was a significant increase in the AIP monitored in Group III (CIMT more than 0.5 mm) when compared to Group II (CIMT less than 0.5 mm) and the control group that also displayed a positive correlation with CIMT. These findings were in agreement with many studies that reported a high predictive value of AIP for the development of atherosclerosis [7,41].

In accordance with Kattamis et al. [42], the current study revealed the elevation in oxidative damage in thalassemia by detecting that MDA significantly increased in β-TM patients while SOD and catalase significantly decreased compared to controls, which was consistent with earlier research. It has been hypothesized that excessive amounts of denatured α or β globin chains, intracellular iron overload, and low concentrations of normal hemoglobin contribute to oxidative damage in β-TM.

The transcription factor (FOXO1), which controls the pathway(s) regulating erythroid maturation and the levels of oxidative stress in murine erythropoiesis, is involved in the intracellular response to oxidative stress in erythropoiesis. In abnormal erythropoiesis, which is characterized by elevated ROS levels, such as inβ-TM, activation of FOXO1 has been proposed as a protective mechanism [43]. FOXO1 translocates into the nucleus in response to iron-overload-induced oxidative stress. FOXO1 defends the cell against low to moderate oxidative stress. When oxidative stress around the tissue is increased, FOXO1 promotes cell death and apoptosis [44]. FOXOs inhibition, on the other hand, led to a decrease in oxidative stress resistance and an increase in ROS levels, demonstrating that FOXOs play a crucial role in ROS resistance. The FOXO1 effect is mediated by the up-regulated transcription of ROS scavenging enzymes, superoxide dismutase, and catalase [45].

The data obtained from the current study show that FOXO1 expression levels differ significantly in the PBMCs of β-TM patients and healthy controls, with a more significant increase in CIMT in the more than 0.5 mm group. The association between FOXO1 expression level and features of atherosclerosis has been reported by several studies [46,47]. Puthanveetil et al. [48] found that the deletion of the FOXO1 gene from cardiac tissue leads to the protection of the heart against cardiomyopathy. They also revealed that down-regulation of the FOXO1 gene in the endothelial tissue could arrest the formation of atherosclerosis plaque [47]. Hence, increments of FOXO1 and its gene expression in β-TM patients are suggested to be a marker of atherosclerosis, as FOXO1 deacetylation can lead to atherosclerotic plaque formation due to endothelial tissue dysfunction in arteries. FOXO1 induced vascular apoptosis and atherosclerotic plaque when endothelial cells were exposed to persistent oxidative stress [49]. The assessed FOXO1 relative gene expression elevations reported here represent its effect on oxidative stress, which was positively correlated with increases in CIMT and arterial stiffness.

Furthermore, according to Kitada et al. [50], our results revealed that the level of SIRT1 decreased in the β-TM patients compared to the healthy controls. It was also shown that this reduction was intensified in patients with CIMT of more than 0.5 mm. The SIRT1 has been recognized to have an important role in the improvement of atherosclerotic plaque, inflammation occurring in response to resistance to stress, and aging [50,51]. SIRT1 has a cardioprotective effect and could maintain vascular function against ROS through the deacetylation of numerous cellular targets. SIRT1 contributes to maintaining vascular hemostasis through the SIRT1/FOXO1 axis. The relationship between FOXO1 and SIRT1 suggests that oxidative stress resistance has evolved through time by deacetylating FOXO1 and decreasing the oxidative stress response, meanwhile, Sirt1 improves FOXO1 DNA binding capacity [52]. Endothelial overexpression of SIRT1 could reduce oxidative–induced aging through the eNOS-dependent signaling pathway [21]. In a previous study, it was found that SIRT1 blocked apoptosis through FOXO1 deacetylation in vascular adventitial fibroblasts [18].

Conceivably, the participation of SIRT1 retrospective to the oxidative stress-mediated posttranslational modifications discovered in SIRT1 may represent the adaptive response to environmental stress under acute conditions. These modifications, however, can increase the deleterious magnitude of atherogenesis influencing CIMT, arterial stiffness, and coronary artery disease in cases of chronic oxidative/environmental stress [53]. When iron overload causes oxidative stress and results in lower levels of catalase and SOD in both β- TM groups compared to controls, this may be related to SIRT1’s role in deacetylating FOXOs and modulating the response to oxidative stress [54]. Additionally, a reduced SIRT1 expression can exacerbate the acetylation of the Atg5 protein, a crucial component of the autophagy mechanism, which further controls the production of foam cells in THP-1 macrophages that are stimulated by ox-LDL [55].

A more conclusive outlook was found herein identifying a positive correlation between CIMT, β arterial stiffness, and HSP 72 that appeared in agreement with earlier reports [56,57]. Bobryshev et al. [58] found that HSP70 was expressed even in dendritic cells in the arterial wall and indicated that elevated HSPs in plaque cells, particularly macrophages, were more stressed within the depth of the atheroma, especially in association with necrosis. In contrast, Suzuki et al. [59] stated that patches of smooth muscle cells were observed in the most complex plaques but without consistent association with necrosis or increased HSP70. Such aforementioned relationships emphasize furthermore the importance of assessed HSP 72 increments in association with their inflammatory-induced response to ROS via the impact of plasma ferritin increments that promote atherogenicity in β-TM under study.

In the current study, partial correlation and multiple linear regression revealed that HSP72 and FOXO1 levels were the strongest independent determinants of CIMT in β-TM children. Pockley et al. [60] indicated that circulating Hsp72 levels predict the development of atherosclerosis in subjects with established hypertension. Furthermore, Kazemi Fard et al. [61] stated that FOXO1 acts as a sensitive biomarker that may serve as an independent factor for predicting CAD.

Analysis of the ROC curve found high sensitivity of FOXO1 gene expression and SIRT1 and revealed FOXO1 gene expression to have better sensitivity than SIRT1. FOXO1 and its gene expression as well as SIRT1 and HSP72 were suggested as predictors of early detection of premature atherosclerosis, but as far as our knowledge, our team was the first to prove such results in β-TM patients.

## 5. Conclusions

Based on the findings presented here, patients with β-TM encountered oxidative stress as a result of iron overload, which is a key factor in the emergence of vascular problems. We may be able to better predict cardiovascular disorders in children by understanding the pathophysiology of the atherosclerotic diseases using a combination of clinical criteria, carotid evaluation, and epigenetic markers such as SIRT1/FOXO1. As a result, the data obtained from the current study may provide a hint for the development of crucial management methods in parallel with other procedures for the early detection of people at risk for atherosclerosis to slow the progression of CAD, particularly in children with β-TM.

## Figures and Tables

**Table 1:** Statistical comparisons among all the studied groups as regards all the studied parameters.

Parameters		Groups			ANOVA	
		Group I (*n =* 50) Control	Group II (*n* = 50) CIMT ≤ 0.5 mm	Group III (*n* = 50) CIMT > 0.5 mm	F	*p*-value
Age	Range	6–13	6–12	6–14	0.384	0.682 (ns)
	Mean ± SD	8.700 ± 1.799	8.840 ± 1.777	8.850 ± 1.911		
BMI (kg/m^2^)	Range	18.5–23.6	18.6–24	23–26.5	153.943	<0.001 *
	Mean ± SD	20.908 ± 1.682	21.222 ± 1.646 *	25.5 ± 0.712 *^#^		
CIMT (mm)	Range	0.20–0.39	0.24–0.48	0.51–1.10	296.486	<0.001 *
	Mean ± SD	0.278 ± 0.057	0.380 ± 0.054 *	0.774 ± 0.169 *^#^		
Beta stiffness	Range	2–2.6	3–3.7	4–4.6	668.952	<0.001 *
	Mean ± SD	2.316 ± 0.162	3.418 ± 0.172 *	4.278 ± 0.1345 *^#^		
Ferritin (ng/dl)	Range	28–165	1450–7655	1655–7800	209.315	<0.001 *
	Mean ± SD	60.94 ± 29.860	4327.32 ±1397.42 *	4570.88 ± 1629.94 *^#^		
AIP	Range	0.11–0.30	0.15–0.35	0.46–0.63	720.547	<0.001 *
	Mean ± SD	0.219 ±0.050	0.277 ± 0.047 *	0.552 ± 0.043 *^#^		
SIRT1 (ng/mL)	Range	2.5–4	1.5–3	0.5–1.9	278.359	<0.001 *
	Mean ± SD	3.17 ± 0.53	2.175 ± 0.358 *	1.145 ± 0.371 *^#^		
ANP (pg/mL)	Range	25–67	60–98	105–126	552.919	<0.001 *
	Mean ± SD	44.12 ± 13.17	75.34 ± 11.19 *	113.66 ± 5.42 *^#^		
CPK MB (U/L)	Range	25–126	28–132	29–140	0.040	<0.961
	Mean ± SD	59.24 ± 26.99	59.5 ± 26.99	60.78 ± 33.41		
LDH1 (U/L)	Range	160–200	180- 240	210–280	434.376	<0.001 *
	Mean ± SD	179.38 ±10.016	222.8 ± 12.378 *	258.32 ± 16.918 *^#^		
Hepcidin (ng/mL)	Range	33–42	18–25	13–15	1735.039	< 0.001 *
	Mean ± SD	37.40 ± 3.024	22.30 ± 1.555 *	13.958 ± 0.803 *^#^		
HSP72 (ng/L)	Range	25.20–36.15	48.07–184.86	50.73–200.65	57.608	<0.001 *
	Mean ± SD	30.128 ± 3.562	92.349 ± 42.561 *	105.845 ± 49.223 *^#^		
SOD (U/L)	Range	6.5–8.5	5.6–7	3–4.25	1111.727	<0.001 *
	Mean ± SD	7.48 ± 0.572	6.51 ± 0.428 *	3.50 ± 0.263 *^#^		
Catalase (U/L)	Range	32–35	29–33.2	11.5–13.6	8836.11	<0.001 *
	Mean ± SD	33.614 ± 0.989	30.046 ± 0.962 *	12.398 ± 0.540 *^#^		
MDA (Nmol/mL)	Range	1.25–3.5	4–8.1	5.5–8.54	464.527	<0.001 *
	Mean ± SD	2.236 ± 0.75	6.258 ± 1.047 *	7.365 ± 0.832 *^#^		
FOXO1 (Ng/mL)	Range	3–4.9	6–8.3	8.5–15.7	1541.81	<0.001 *
	Mean ± SD	3.436 ± 0.489	7.22 ± 0.704 *	12.76 ± 1.184 *^#^		
FOXO1 relative gene expression	Range	0.8–1.9	0.9–2.23	2.3–3.4	496.654	<0.001 *
	Mean ± SD	1.155 ± 0.226	1.725 ± 0.277 *	2.702 ± 0.239 *^#^		

Data presented as mean + SD. *p* value was calculated via one-way ANOVA test followed by Tukey’s post hoc test, SPSS computer program. *: significant difference vs. Group I, control group (*p* < 0.001). #: significant difference vs. Group II, CIMT ≤ 0.5 mm. BMI: body mass index; CIMT: carotid intima-media thickness; AIP: atherogenic index of plasma; ANP: atrial natriuretic peptide; CPK MB: creatine phosphokinase; LDH1: lactate dehydrogenase; HSP72: heat shock protein 72; SOD: superoxide dismutase; MDA: malondialdehyde; SIRT1: sirtuin1; FOXO1: forehead box protein 1.

**Table 2:** Pearson’s and partial correlations between CIMT and among all studied groups as regards all the studied parameters.

Pearson’s Correlations			Partial Correlation Coefficient	
	CIMT (mm)			
	r	*p*-Value	r′	*p*-Value
BMI	0.716	<0.001 *	−0.019	0.829
AIP	0.851	<0.001 *	0.095	0.270
Hepcidin (ng/mL)	−0.589	<0.001 *	0.062	0.470
ANP (pg/mL)	0.825	<0.001 *	0.079	0.355
CPK MB (U/L)	−0.013	0.875	−0.150	0.078
LDH1 (U/L)	0.765	<0.001 *	−0.039	0.646
Hsp72 (ng/mL)	0.365	<0.001 *	−0.306	<0.001 *
SIRT1(ng/ml)	−0.760	<0.001 *	0.023	0.798
SOD (U/L)	−0.863	<0.001 *	−0.047	0.583
Catalase (U/L)	−0.887	<0.001 *	−0.106	0.216
MDA (Nmol/mL)	0.660	<0.001 *	0.014	0.869
FOXO1 (ng/mL)	0.876	<0.001 *	0.282	0.001 *
FOXO1 relative gene expression	0.835	<0.001 *	0.097	0.256

r: correlation coefficient; r′: partial correlation coefficient; *p*: *p*- value (significant < 0.05); *: according to Mann–Whitney’s U-test.

**Table 3:** Multiple linear regression analysis using biologically important variables as independent variables and CIMT as the dependent variable.

Variables	Unstandardized Estimate Parameter		β	Standardized Estimate Parameters		95.0% C.I. for Odd	
	B	S.E.		t	*p*-Value	Lower	Upper
BMI	−0.004	0.006	−0.036	−0.585	0.560	−0.015	0.008
HSP72	−0.001	0.000	−0.179	−30.711	0.000	−0.001	0.000
SIRT1	0.022	0.021	0.085	10.069	0.287	−0.019	0.063
SOD	−0.007	0.019	−0.049	−0.356	0.722	−0.044	0.031
Catalase	−0.007	0.004	−0.260	−10.520	0.131	−0.015	0.002
LDH1	−0.001	0.000	−0.071	−10.965	0.051	−0.001	0.000
ANP	00.000	0.001	−0.003	−0.035	0.972	−0.001	0.001
HEPCIDINE	0.001	0.001	0.124	10.179	0.240	−0.001	0.003
FOXO1	−0.002	0.001	−0.180	−20.131	0.035	−0.003	0.000
FOXO1 gene expression	0.038	0.010	0.625	30.956	0.000	0.019	0.057
Constant	0.389	0.278		10.402	0.163	−0.160	0.939

B: regression coefficient, S.E: standard error, β: standard coefficient, CI: confidence interval.

## Data Availability

All data that support the findings of the current study are available upon reasonable request.
